# Supplementary material for: Incidence and Severity of Community- and Hospital-Acquired Hyponatremia in Pediatrics
Source: J Clin Med. 2022 Dec 19;11(24):7522. doi: 10.3390/jcm11247522 (PMC9782113; doi:10.3390/jcm11247522)
Supplement: Supplementary file 1 [file jcm-11-07522-s001.zip › jcm-2052359-supplementary.pdf]

**Supplementary Table S1:** Diagnostic groups, ICD-9 and ICD-10 disease and procedural codes.

| Diagnostic Groups | Diagnosis and Codification | | |
|---|---|---|---|
| **Infections** | **Diagnosis** | **ICD9** | **ICD10** |
| | Septicemia | 038, 790.7 | A39, A40, A41, A49, R78.81 |
| | Meningitis | 047, 094, 320–322, 324 | A17, A87, G00, G03 |
| | Encephalitis | 063, 064, 052.0, 054.3, 058.2, 323 | G04, G05, A86 |
| | Pneumonia | 480–488 | J12–J18 |
| | Upper respiratory tract infections | 460–464, 470–478 | J00–J06, J30–J39 |
| | Bronchiolitis | 466.1 | J21 |
| | Other respiratory infections | 465.8, 465.9 | J22 |
| | Viral infections | 050–075, 079.99 | A08, B00–B34, B97 |
| | Diarrhea | 001–009, 787.91 | A09, R19.7 |
| | Urinary tract infections | 595, 599 | N30, N39 |
| | Pyelonephritis | 590 | N10–N11, N13.6 |
| | Cellulitis | 376.01, 528.3, 681, 682, 684 | L01–L08, H05 |
| | Osteomyelitis * | 730 | M86 |
| | Myocarditis $ | 422, 429.0 | I40, I41 |
| | Abscess | 475, 510, 513, 556, 566 | J36, J85, K61 |
| | Otitis Media | 380–382 | H65, H66, H70 |
| | Toxic Shock Syndrome | 040.82 | A48 |
| | Steven Johnson Syndrome | 695.13, 695.14 | L51.3, L51.4 |
| | Miscellaneous | 780.6, 996.67,998.59 | N45.3, R50.9, T81.4 |
| Respiratory disorders | Diagnosis | ICD9 | ICD10 |
| | Chronic diseases of upper airway | 470–478 | J32–35, J37 |
| | Asthma, Bronchitis | 490–496 | J40, J42, J45.901 |
| | Pneumothorax, Other diseases of lung and respiratory system | 510–519 | J93, J95, J98 |
| | Acute respiratory failure | 518.81 | J96 |
| | Acute bronchospasm | 519.11 | J98.01 |
| | Symptoms of respiratory system- apnea, dyspnea, stridor, cough | 786.1, 786.2, 786.03, 786.0 | R04, 06, 07, 09 |
| | Foreign body in upper airways | 933 | T16–T17 |
| Cardiovascular disorders | Diagnosis | ICD9 | ICD10 |
| | Hypertension | 401 | I10–I16 |
| | Inflammatory pericardial diseases | 420, 423 | I30, I31, I32 |
| | Cardiac dysrthymias | 427, 785 | I44– I49, R00 |
| | Kawasaki's disease | 446.1 | M30.3 |
| | Venous thrombosis | 453 | I82 |
| | Syncope | 780.2 | R42, R55 |
| | Chest pain | 786.5 | R07.2, R07.8 |
| | Congenital anomalies of heart | 745–747 | Q22–Q25 |
| Gastrointestinal disorders | Diagnosis | ICD9 | ICD10 |
| | Supernumerary tooth | 520.1 | K00.1 |
| | Dental caries, Chronic gingivitis | 521.0, 523.1 | K02, K05.1 |
| | Aphthous ulcers | 528.2 | K12.0 |
| | Gastroesophageal reflux | 530.81 | K21 |
| | Gastritis | 535 | K29.0 |

| | | | |
|---|---|---|---|
| | Crohn's disease, Ulcerative colitis, Irritable colon | 555, 556 | K50, K51, K58 |
| | Non-infectious gastroenteritis and colitis | 558 | K52 |
| | Constipation, Fecal impaction | 564.0, 560.32 | K59.0, K56.41 |
| | Malabsorption | 579 | K90 |
| | Other disorders of biliary tract | 576 | K87 |
| | Vomiting | 787.0 | R11 |
| | Abdominal pain | 789.0 | R10 |
| | Foreign body in digestive tract | 935–938 | T18 |
| | Mesenteric adenitis | 289.2 | I88.0 |
| | Hematemesis, Malena | 578 | K92.0, K92.1 |
| Genito-urinary disorders | Diagnosis | ICD9 | ICD10 |
| | Acute and Chronic glomerulonephritis | 580, 582 | N00–N03 |
| | Renal failure | 584 | N17–N19 |
| | Nephrotic syndrome | 581 | N04 |
| | Renal colic | 788.0 | N20, N23 |
| | Follicular cyst of ovary | 620 | N83.0 |
| | Hematuria | N/A | R31 |
| | Epididymo-orchitis | 604 | N45.3 |
| Neurological disorders | Diagnosis | ICD9 | ICD10 |
| | Epilepsy | 345 | G40 |
| | Migraine | 346 | G43 |
| | Convulsions | 780.3 | R56 |
| | Headache | 339, 784.0 | G44, R51 |
| | Benign Intracranial Hypertension | 348.2 | G93.2 |
| | Ataxia | 334 | G32.81, R27.0 |
| | Transverse myelitis | 341.2 | G37.3 |
| | Hemiplegia | 342 | G81 |
| | Cerebral palsy | 343 | G80 |
| Endocrine and Metabolic disorders | Diagnosis | ICD9 | ICD10 |
| | Diabetes Mellitus | 250 | E10 |
| | Precocious puberty | 259.1 | E30.1 |
| | Short stature | 783.43 | R62.52 |
| | Hypothyroidism | 243, 244 | E03 |
| | Acidosis, Alkalosis | 276.2, 276.3 | E87.2, E87.3 |
| | Hypoglycemia | 251.2 | E16.1, E16.2 |
| Musculoskeletal disorders | Diagnosis | ICD9 | ICD10 |
| | Juvenile Idiopathic Arthritis | 714.3 | M08 |
| | Other nonspecific arthritis | 716 | M07, M13 |
| | Torticollis | 723.5 | M43.6 |
| | Synovitis | 727.0 | M65.9 |
| | Tietze's syndrome | 733.6 | M94.0 |
| | Myalgia | 729.1 | M79.1 |
| Trauma (Surgical) | Diagnosis | ICD9 | ICD10 |
| | Skull and facial bone fractures | 800–802 | S02, S09 |
| | Fracture of Upper Limb | 810–819 | S41–S43, S51, S52, S61, S62 |
| | Fracture of Lower Limb | 820–829 | S71, S72, S81, S82, |
| | Dislocation | 830–839 | S73, S83, S91, S92 |

| | | | |
|---|---|---|---|
| | Intracranial injury excluding skull fracture | 851–854 | S06.4 to S06.9 |
| | Internal injury to chest, abdomen, pelvis | 860–869 | S20, S27, S29, S30, S39 |
| | Open wound of head | 873 | S01 |
| | Open wound of upper limb | 880–884 | S41 |
| | Open wound of back, buttock, genitals, unspecified | 876–879 | S31 |
| | Effects of foreign body entering through orifices | 930–939 | T15–T19 |
| Surgical | Diagnosis | ICD9 | ICD10 |
| | Tonsillectomy without and with adenoidectomy | 28.2, 28.3 | 0CTP, 0CTQ |
| | Myringoplasty | 19.4 | 09Q7, 09Q8 |
| | Squint surgery | 15.1, 15.2, 15.3, 15.4 | 08BL, 08BM |
| | Incision of perianal abscess | 49.01 | 0HB8, 0HB9 |
| | Appendectomy | 47.0, 47.11 | 0DTJ |
| | Hydrocele repair | 61.2 | 0V96, 0V97 |
| | Unilateral orchiectomy | 62.3 | 0VPD |
| | Orchidopexy | 62.5 | 0VQ9, 0VQB, 0VQC, 0VS9, 0VSB |
| | Circumcision | 64.0 | 0VTT |
| | Open reduction and internal fixation of fracture | 79.2, 79.3, 79.5, 79.6, 79.8 | 0PP, 0PQ, 0PS, 0QQ, 0QR, 0QS, |
| | Excision and division of bones, other operations on bone | 77, 78 | 0QB |
| | Arthroscopy | 80.2 | 0SQC4ZZ, 0SQD4ZZ |
| | Repair of cruciate ligaments | 81.45 | 0SC4ZX, 0SBD4ZX |
| | Inguinal hernia repair | 53.0, 53.1 | 0YQ5, 0YQ6, |
| | Dental extraction | 23.1 | 0CDW, 0CDX |
| | Endoscopy | 45.11, 45.21,45.23, 45.24 | 0DJ0, 0DJ6, 0DJD |
| Skin and Subcutaneous disorders | Diagnosis | ICD9 | ICD10 |
| | Hemangioma | 228 | D18 |
| | Atopic dermatitis | 691–693 | L20–L22 |
| | Urticaria | 708.1, 708.9 | L50.1, L50.9 |
| Mental Health disorders | Diagnosis | ICD9 | ICD10 |
| | Substance abuse- alcohol, cannabis, opiates | 305.0, 305.2, 305.5 | F10.12, F11.1, F12.1 |
| | Psychosis | 298 | F23, F29 |
| | Anorexia nervosa | 307 | F50.0 |
| | Suicidal ideation | E950, E956 | R45.851 |
| | Anxiety disorders | 300.0 | F41, F42, F43 |
| Hematological and Immune disorders | Diagnosis | ICD9 | ICD10 |
| | Nutritional anemias | 280–281 | D50–D53 |
| | Hemolytic anemias | 282–283 | D55–D59 |
| | Immune thrombocytopenic purpura | 287.31 | D69.3 |
| | Hereditary hypogammaglobulinemia | 279.00 | D80.0 |
| Allergy and Toxins | Diagnosis | ICD9 | ICD10 |
| | Allergic purpura | 287.0 | D69.0 |
| | Allergic urticaria | 708.0 | L50.0 |

| | | | |
|---|---|---|---|
| | Poisoning by drugs, medicinal and biological substances | 960–979 | T36–T50 |
| | Toxic effects of non-medicinal substances | 980.0 | T51–T65 |
| | Anaphylactic reactions | 995 | T78.0 |
| Obstetric/Pregnancy andChildbirth | Diagnosis | ICD9 | ICD10 |
| | Abortion | 632, 637 | O03 |
| | Preterm labor | 644 | O60 |
| | Normal labor | 650 | O80 |
| | Forceps delivery | 669.5 | O66.5 |
| Eye disorders | Diagnosis | ICD9 | ICD10 |
| | Strabismus | 378 | H49, H50 |
| | Papilledema, not specified | 377.0 | H47.1 |
| | Diplopia | 368.2 | H53.2 |
| | Nystagmus | 379.5 | H55 |
| Miscellaneous disorders | Diagnosis | ICD9 | ICD10 |
| | Observation and evaluation for suspected condition not found | V71 | Z01 |
| | Special investigation and examinations | V72 | Z02 |
| | Medical observation condition ruled out | N/A | Z03 |
| | Examination and observation for other reasons | N/A | Z04 |
| Nonsurgical trauma | Diagnosis | ICD9 | ICD10 |
| | Concussion | 850 | S06.0, S09.9, |
| | Sprains and Strains | 840–848 | S43.4, S49.8, S53.4, S59.1, S63.5–S63.9, S73.1, S79.9, S83.3–S83.9, S93.4, S99.9, |
| | Contusion with intact skin | 920–924 | S20, S30, S40, S50, S60, S70, S80, S90 |
| | Burns medically managed | 942–946 | T20–T25 |
| | Unspecified injury | 959 | T14 |
| | Child abuse | 995.5 | T74 |

"Nonsurgical trauma" corresponds with "Trauma" in Table 1 and "Surgical trauma" is included in "Surgical" category in Table 1.
